# *Streptococcus pneumoniae* GAPN is a key metabolic player necessary for host infection

**DOI:** 10.1002/pro.5253

**Published:** 2024-12-11

**Authors:** Eunjeong Lee, Anthony Saviola, Shaun Bevers, Jasmina S. Redzic, Sean P. Maroney, Steven Shaw, Emily Tamkin, Sam Fulte, Travis Nemkov, Nancy Meyer, Angelo D'Alessandro, Kirk C. Hansen, Sarah E. Clark, Elan Eisenmesser

**Affiliations:** ^1^ Department of Biochemistry and Molecular Genetics, School of Medicine University of Colorado Anschutz Medical Campus Aurora Colorado USA; ^2^ Department of Otolaryngology, School of Medicine University of Colorado Anschutz Medical Campus Aurora Colorado USA; ^3^ Pacific Northwest Cryo‐EM Center Oregon Health and Science University Portland Oregon USA

**Keywords:** cryo‐EM, GAPN, glycolysis, metabolism, structure

## Abstract

*Streptococcus pneumoniae* (*S. pneumoniae*) employs various metabolic pathways to generate nicotinamide adenine dinucleotide phosphate (NADPH), which is essential for redox balance, fatty acid synthesis, and energy production. GAPN, a non‐phosphorylating glyceraldehyde‐3‐phosphate dehydrogenase, plays a role in this process by directly reducing NADP^+^ to NADPH, effectively contributing to glucose metabolism. However, its relative importance for *S. pneumoniae* metabolism and infection has remained unknown. Here, we performed a comprehensive characterization of *S. pneumoniae* GAPN through kinetic assays, isothermal titration calorimetry (ITC), cryo‐EM, mass spectrometry, and infection assays. Despite its structural similarities to its homologues in other species, *S. pneumoniae* GAPN exhibits negative cooperativity with respect to its substrate, glyceraldehyde‐3‐phosphate (G3P), suggesting a unique regulatory mechanism. Our results demonstrate that GAPN knockout leads to significant metabolic reprogramming, including increased glycogen storage that leads to enhanced fatty acid metabolism. This collectively reduces the ability of *S. pneumoniae* to manage oxidative stress and sustain infection. Our findings highlight GAPN as a critical enzyme for *S. pneumoniae* metabolic balance and suggest that its inhibition could serve as a potential strategy for therapeutic intervention in pneumococcal diseases.

## 1 INTRODUCTION

**FIGURE 1:**
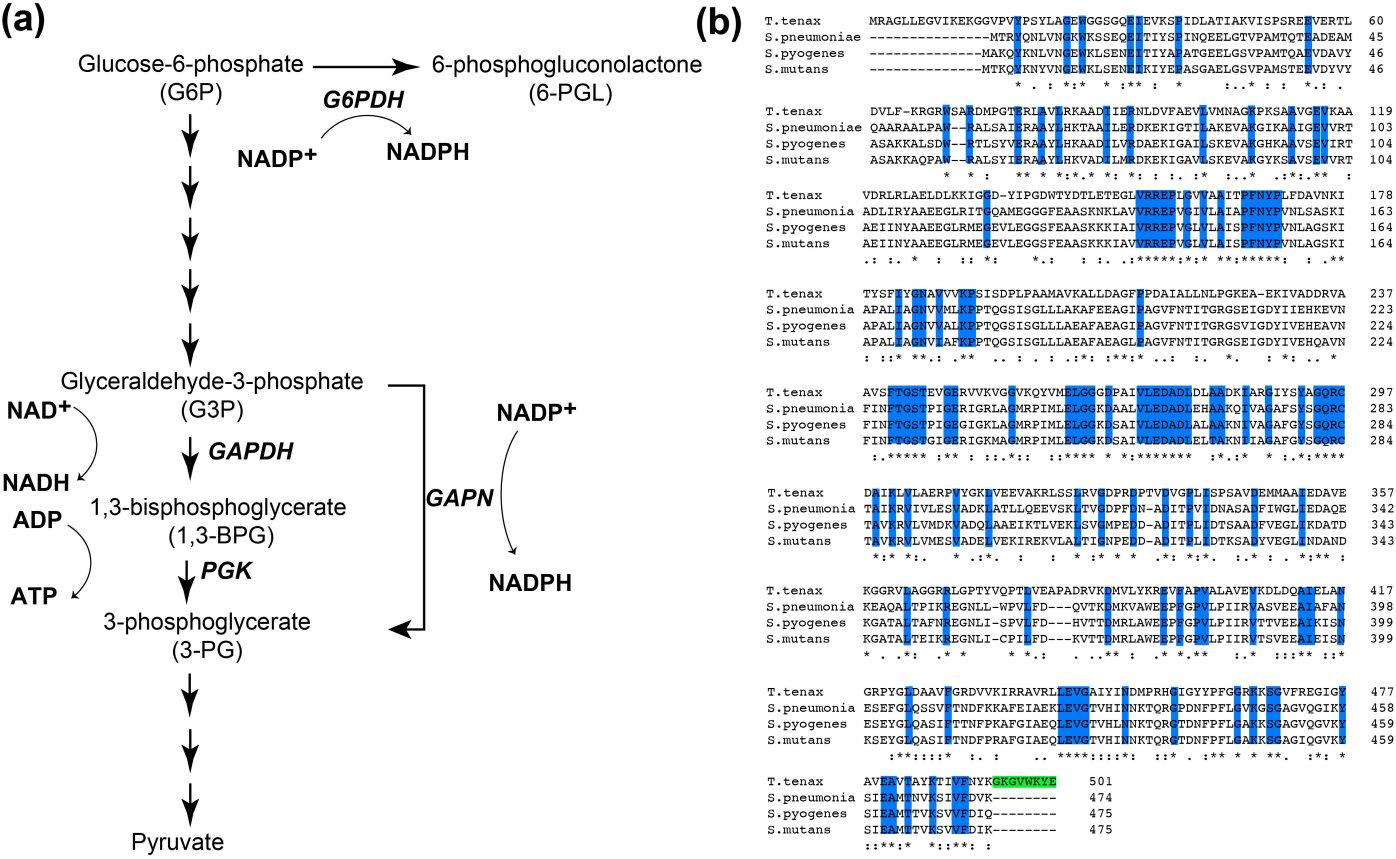
The enzymatic role and sequence comparisons between homologues for GAPN. (a) Schematic of the 10 steps of glycolysis that specifically focuses on GAPDH and PGK. GAPN facilitates the direct oxidation of G3P to 3‐PG using NADP^+^ as a cofactor, bypassing the traditional two‐step process. Additionally, the first step of the PPP shunt of glucose‐6‐phosphate‐dehydrogenase (G6PDH) is shown. (b) Sequence homology of GAPN homologues from *T. tenax* and the three Gram‐positive bacteria, *S. pneumoniae*, *S. pyogenes*, and *S. mutants*. Completely conserved residues are highlighted (blue) and the eight‐residue extension in *T. tenax* (green). Additional elements of conservation are delineated below these sequences as completely conserved residues (*), along with mostly conserved residues (:), and partially conserved residues (.)

Despite their apparent simplicity compared to multicellular organisms, the metabolic complexities of prokaryotes continue to unfold (Willenborg and Goethe 2016). Over a century after the identification of infectious bacteria, we are still unraveling the mechanisms that provide their most fundamental requirements of life. Among these essentials are energy production and redox regulation, facilitated by molecules such as nicotinamide adenine dinucleotide phosphate (NADPH). Bacteria employ diverse mechanisms to generate NADPH, which can vary between different bacterial species but also among strains within the same species. For instance, certain prokaryotes and archaea possess a non‐phosphorylating glyceraldehyde‐3‐phosphate dehydrogenase, called GAPN (Spaans et al. 2015). GAPN reduces NADP^+^ to NADPH, effectively circumventing glycolytic reactions of glyceraldehyde‐3‐phosphate dehydrogenase (GAPDH) and phosphoglycerate kinase (PGK) by directly converting glyceradehyde‐3‐phosphate (G3P) into 3‐phosphoglycerate (3‐PG) (Figure 1a). This bypasses an ATP‐generating step of glycolysis by skipping the step catalyzed by phosphoglycerate kinase (PGK). Several opportunistic pathogens, including various streptococcal species, encode GAPN in their genomes. However, due to the unique metabolic pathways available within these different streptococci species, the precise dependence on GAPN could potentially differ due to the presence of other enzymes that can also recycle NADP^+^ back to NADPH (Spaans et al. 2015). These differences include the use of glucose‐6‐phosphate dehydrogenase (G6PDH) that comprises the first and rate‐limiting step of the oxidative phase of the pentose phosphate pathway (PPP), and isocitrate dehydrogenase that, like anucleate mammalian red blood cells, is selective for reduction of NADP^+^ in bacteria (Willenborg and Goethe 2016). *Streptococcus pyogenes* (*S. pyogenes*) and *Streptococcus mutans* (*S. mutans*) do not comprise a fully developed PPP, while *Streptococcus pneumoniae* (*S. pneumoniae*) does in fact comprise a full PPP. Only *S. mutans* encodes its own isocitrate dehydrogenase. To date, the critical role of GAPN in bacteria metabolism has only been shown to be important in *S. pyogenes* infection (Eisenberg et al. 2022) and thus, further studies are needed to assess the role of GAPN in other streptococci. Our studies here have revealed a surprisingly critical role of *S. pneumoniae* GAPN that leads to rewiring of cellular metabolism and a markedly reduced virulence.

The structure and biochemical properties of several GAPN homologues have been determined. These include GAPN from the archaeon *Thermoproteus tenax* (*T. tenax*) (Lorentzen et al. 2004; Pohl et al. 2002), and from two streptococci, *Streptococcus mutans* (*S. mutans*) (Cobessi et al. 1999; Cobessi et al. 2000) and *S. pyogenes* (Eisenberg et al. 2022). Both the sequences and structures of these homologues indicates that they are closely related to the aldolase family and likely evolved through a lineage distinct from that of GAPDH (Habenicht et al. 1994). Interestingly, *T. tenax* GAPN is allosterically activated by several metabolites, while *S. pyogenes* GAPN and *S. mutans* GAPN are directly inhibited by erythrose 4‐phosphate (E4P) binding to their active sites (Eisenberg et al. 2022; Lorentzen et al. 2004). However, *T. tenax* GAPN shares only 35% identity with its streptococci homologues, while *S. pyogenes and S. mutans* GAPNs exhibit 85% identity. *Streptococcus pneumoniae* GAPN shares less homology with its streptococcal homologues with 70% identity to both *S. pyogenes and S. mutans* GAPNs. The sequence comparisons of all four of GAPN homologues also indicate that *T. tenax* GAPN comprises both N‐terminal and C‐terminal extensions (Figure 1b). As it is the C‐terminal extension within *T. tenax* GAPN that orchestrates an allosteric response to effectors (Lorentzen et al. 2004; Pohl et al. 2002), none of the streptococcal homologues were expected to exhibit such cooperativity. However, although our structural studies reveal a conserved architecture, our biochemical studies have revealed that substrate binding is negatively allosteric.

Here, we have cloned and characterized *S. pneumoniae* GAPN for the first time to fully evaluate its biochemical, structural, and biological details that include infection. We have applied UV‐kinetics, isothermal titration calorimetry (ITC), cryo‐EM, mass spectrometry, and infection assays to fully characterize *S. pneumoniae* GAPN. Beyond revealing that *S. pneumoniae* GAPN is negatively allosteric for its substrate, our data suggest that GAPN is central to maintaining metabolic balance within the bacterial cell. A knockout of *S. pneumoniae* GAPN not only impairs the ability to manage redox balance via NADPH recycling but also forces a metabolic shift towards glycogen storage and enhanced lipid metabolism. This reprogramming appears to render *S. pneumoniae* more susceptible to oxidative stress and less capable of sustaining infection, highlighting GAPN as a potential target for therapeutic intervention in pneumococcal diseases. The observed metabolic changes underscore the complexity of bacterial adaptation to genetic perturbations and suggest that GAPN plays a broader role in bacterial pathogenesis than previously understood.

## 2 RESULTS

### 2.1 *Streptococcus pneumoniae* GAPN exhibits moderate catalytic efficiency measured by steady‐state kinetics and is inhibited by erythrose‐4‐phosphate

**FIGURE 2:**
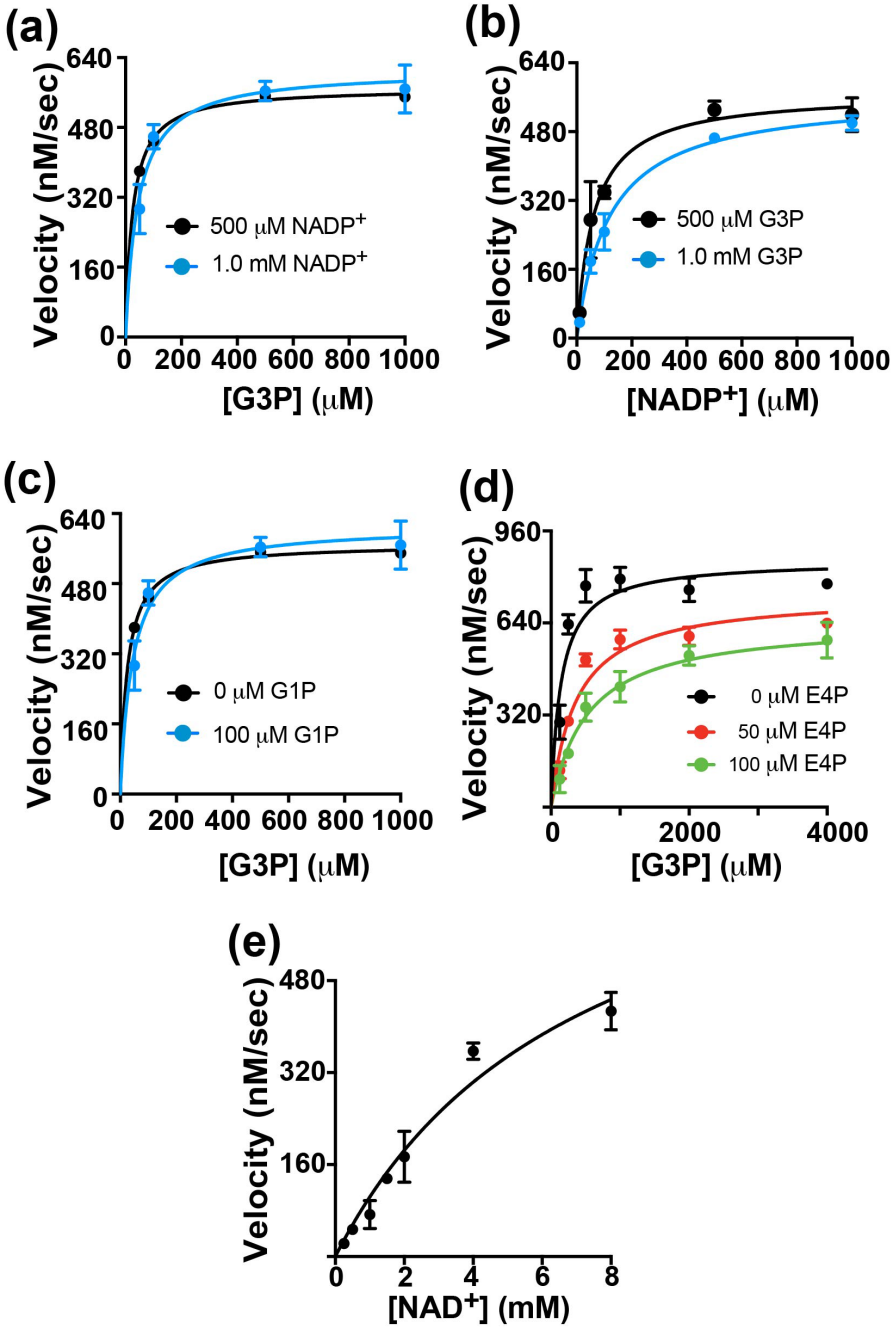
Kinetic characterization of *S. pneumoniae* GAPN. (a) Michaelis–Menten kinetics of GAPN with varying G3P and NADP^+^ fixed at 500 μM and 1 mM G3P. (b) Michaelis–Menten kinetics of GAPN with varying NADP^+^ and G3P fixed at 500 μM and 1 mM G3P. (c) Michaelis–Menten kinetics of GAPN with varying G3P, NADP^+^ fixed at 500 μM, and fixed G1P at either 0 and 100 μM G1P. (d) Michaelis–Menten kinetics of GAPN with varying G3P, NADP^+^ fixed at 500 μM, and fixed E4P at either 0, 50, and 100 μM G1P. (e) Michaelis–Menten kinetics of GAPN with varying NAD^+^ and G3P fixed at 500 μM. Extracted kinetic constants are reported in Table 1 with all UV‐kinetics data collected at 25°C and monitored at 340 nm. All points and uncertainties are based on the averages and standard deviations of three replicates.

Recombinantly purified *S. pneumoniae* GAPN exhibits similar kinetic parameters to those previously measured for *S. mutans* GAPN (Marchal et al. 2000; Marchal and Branlant 2002; Pailot et al. 2006), reported here (Table 1 and Figure 2a,b). Recombinant *S. pneumoniae* GAPN was used to monitor the conversion of G3P and NADP^+^ to 3‐PG and NADPH through standard Michaelis–Menten UV‐kinetics assays. G3P and the oxidized coenzyme, NADP^+^, were assayed at two saturating concentrations of the other with largely similar results, suggesting the fixed concentrations were indeed saturating that is consistent with their fit Michaelis constants (Table 1). *Streptococcus pneumoniae* GAPN catalytic turnover, *k*_cat_, was 14 and 25 s^−1^ at pH 7.5 and pH 8.5, respectively. These values are similar to *k*_cat_ measured for *S. mutans* GAPN of 60 s^−1^ (Marchal and Branlant 2002) but significantly slower than for phosphorylated human GAPDH of 200 s^−1^ (Chaikuad et al. 2011), both measured at pH 8.5. Catalytic efficiencies are relatively high for *S. pneumoniae* GAPN studied here (*k*_cat_/*K*_*M*_ ~ 10^6^ M^−1^ s^−1^), which is also consistent with other GAPN homologues as well (Chaikuad et al. 2011; Ito et al. 2012).

**TABLE 1:** Kinetic parameters of *S. pneumoniae* GAPN from UV‐kinetics.

	*K*_*M*_	*V*max	*k*_cat_	KI (E4P)
G3P (500 μM NADP^+^)^a^	81 ± 21 μM	560 ± 40 nM s^−1^	14.0 ± 1.0 s^−1^	‐
G3P (1.0 mM NADP^+^)^a^	88 ± 17 μM	580 ± 30 nM s^−1^	14.5 ± 0.7 s^−1^	‐
NADP^+^ (500 μM G3P)^a^	101 ± 11 μM	572 ± 25 nM s^−1^	14.3 ± 0.4 s^−1^	‐
NADP^+^ (1.0 mM G3P)^a^	119 ± 12 μM	570 ± 20 nM s^−1^	14.2 ± 0.6 s^−1^	‐
NAD^+^ (1.0 mM G3P)^b^	7.2 ± 2 mM	8.5 ± 1.4 mM s^−1^	0.4 ± 0.1 s^−1^	‐
G3P (500 μM NADP^+^)^c^	143 ± 25 μM	870 ± 40 nM s^−1^	21.8 ± 0.9 s^−1^	25.7 ± 7.2 μM

^a^ pH 7.5 using 40 nM GAPN.

^b^ pH 8.5 using 20 μM GAPN.

^c^ pH 8.5 using 40 nM GAPN.

The potential for allosteric activation and direct active site inhibition was studied for *S. pneumoniae* GAPN. Allosteric activation has been observed in *T. tenax* GAPN through multiple metabolites that include G1P, F6P, AMP, and ADP (Lorentzen et al. 2004), yet no such activation was identified in *S. pneumoniae* GAPN illustrated here with G1P (Figure 2c). This is not surprising, considering the allosteric metabolite binding site is only conserved in archaea GAPNs (Lorentzen et al. 2004), which includes an eight‐residue extension that contribute to the tetramerization domain through the C‐terminus that is absent in streptococci GAPNs (Figure 1b, green). In contrast, E4P potently inhibits *S. pneumoniae* GAPN (Figure 2d). This is consistent with recent findings showing E4P also inhibits *S. pyogenes* GAPN (Eisenberg et al. 2022). For *S. pneumoniae* GAPN, our data were fit with an inhibition constant of 25 μM, which is the first reported affinity of GAPN for E4P to our knowledge.

We also tested NAD^+^ as a coenzyme for *S. pneumoniae* GAPN, considering that many NADP^+^‐dependent enzymes can utilize both coenzymes that include isocitrate dehydrogenase (Willenborg and Goethe 2016). To observe turnover using NAD^+^ as a coenzyme, 500‐fold more of GAPN was necessary than that using the preferred NADP^+^ coenzyme (Table 1 and Figure 2e). NAD^+^ turnover was 0.4 s^−1^, which is 50‐fold slower than that with NADP^+^ of 21 s^−1^ at the same pH. Nonetheless, this is likely a biologically relavent finding, considering that NAD^+^ concentrations are known to be much higher than NADP^+^ concentrations in most bacteria (London and Knight 1966). Furthermore, GAPN expression has recently been shown to be coupled to high NADH concentrations in a complicated redox regulatory mechanism in *S. pneumoniae* (Afzal et al. 2018). These findings, coupled with recent findings, suggest that both coenzymes are likely reduced in vivo by GAPN.

### 2.2 Thermodynamics reveal slow binding and release and an allosteric mechanism for *S. pneumoniae* GAPN

**FIGURE 3:**
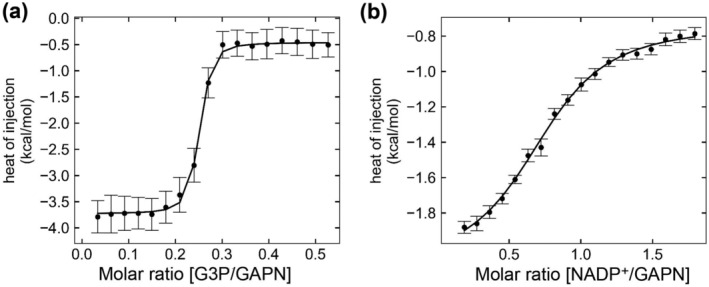
Thermodynamic characterization of *S. pneumoniae* GAPN. (a) ITC of GAPN with varying G3P. (b) ITC of GAPN with varying NADP^+^. Points and error bars were determined from an average of three ITC titrations.

We used ITC to measure the binding affinity and thermodynamic parameters for *S. pneumoniae* GAPN (Table 2 and Figure 3). Unlike kinetic studies presented above that reveal similar Michaelis–Menten constants (*K*_*M*_) values for both NADP^+^ and the G3P substrate, there is a large difference between their dissociation constants (*K*_*D*_). Specifically, the *K*_*D*_ for G3P is 53 nM that is three orders tighter than NADP^+^ that is 51 μM. Based on both the kinetics described above and thermodynamics presented here, we can estimate both the off‐rate (*k*_off_) and the on‐rate (*k*_on_) for the G3P substrate. Specifically, the nanomolar affinity of G3P suggests that the *k*_off_ is negligible compared to the turnover rate, *k*_cat_. Using the definition of the Michaelis rate constant, we can estimate this relationship in Equation (1),

(1) $$K_{M}=\left(k_{\mathrm{off}}+k_{\mathrm{cat}}\right)/k_{\mathrm{on}}≅k_{\mathrm{cat}}/k_{\mathrm{on}}.$$

**TABLE 2:** Thermodynamic parameters of *S. pneumoniae* GAPN binding from ITC.

	*K*_*D*_	Δ*H*	Δ*S*
G3P	53 ± 36 nM	−6.8 ± 0.6 kcal	10.8 ± 3.5 kcal
NADP^+^	51 ± 32 μM	−2.3 ± 0.8 kcal	12.5 ± 2.8 kcal

Substituting *k*_on_ in terms of *k*_off_ and *K*_*D*_ gives the estimate for *k*_off_ as

(2) $$k_{\mathrm{off}}=\left(K_{D}/K_{M}\right)k_{\mathrm{cat}}.$$

For G3P at pH 7.5, its *K*_*D*_ is 53 nM, its *K*_*M*_ is 88 μM, and the *k*_cat_ is ~14 s^−1^, which amounts to an estimated *k*_off_ ~ 0.0084 s^−1^ and *k*_on_ ~ 162,000 M^−1^ s^−1^. Such relatively slow rates may be explained by the buried nature of the substrate in GAPN that would require conformational changes to facilitate binding and release. Importantly, this slow off‐rate applies to the substrate (G3P) and does not reflect a potential rate‐limiting step coupled to the turnover rate, such as the dissociation of the 3‐PG product.

Potentially the most interesting finding from these ITC studies is that one monomer within each tetramer binds G3P (*n* = 0.25), as opposed to the coenzyme that binds each monomer (*n* ~ 1.0). This finding means that there is negative cooperativity within GAPN, which is also consistent with the relatively poor G3P density expected for a single binding site per tetramer described in the next section for cryo‐EM studies. While this is the first study to identify such an allosteric mechanism within GAPN, there have yet to be any similar ITC studies performed for the other family members. Thus, it remains unknown whether *S. pneumoniae* GAPN is unique in this allosteric mechanism within the larger GAPN family. However, as we show with our structural studies below, *S. pneumoniae* GAPN is virtually identical to that of *S. mutans* GAPN and thus, it is possible that this negatively cooperative mechanism is a conserved feature.

### 2.3 Cryo‐EM structural studies of *S. pneumoniae* GAPN reveal the conserved mechanism of metabolic interactions

**FIGURE 4:**
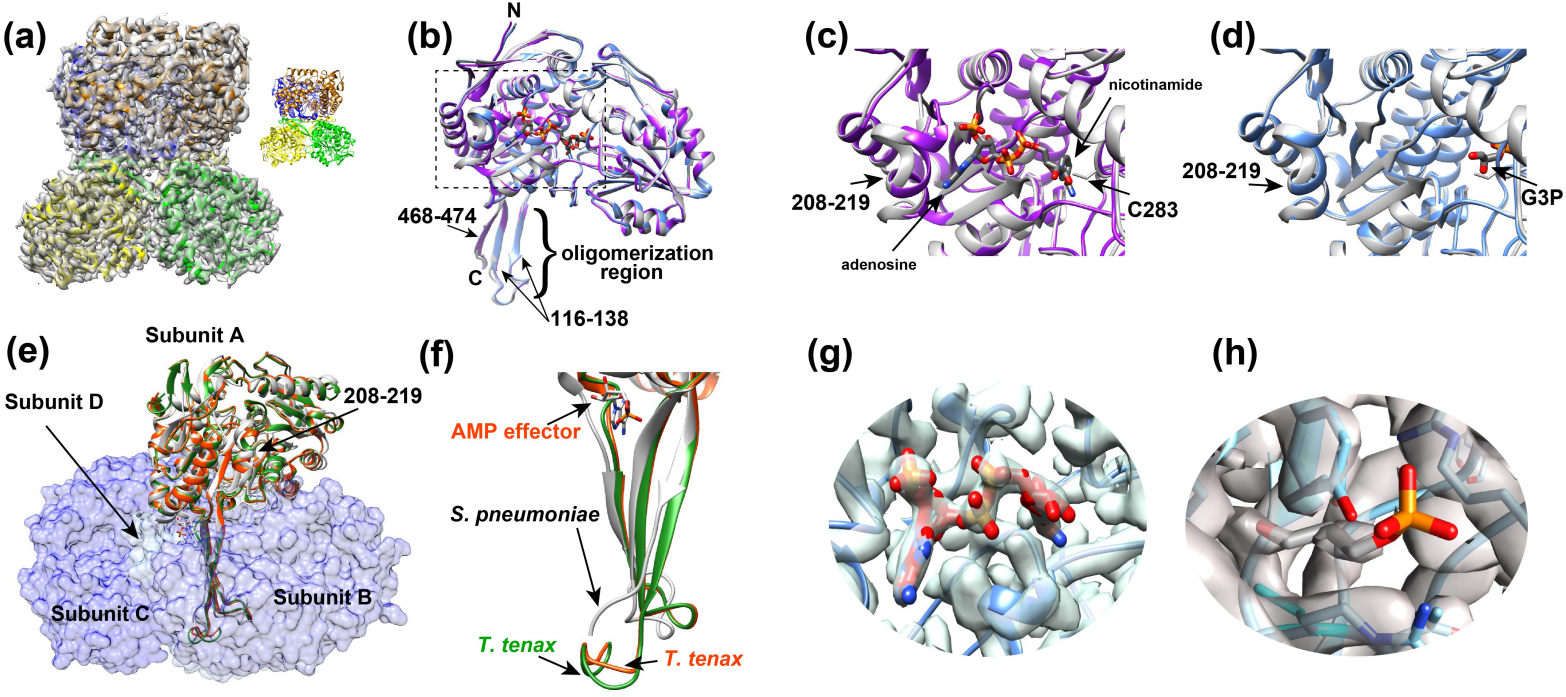
Cryo‐EM studies of *S. pneumoniae* GAPN. (a) Cryo‐EM density and fitted model of apo GAPN with the inset showing the model alone. Four subunits are colored differently (blue, orange, yellow, and green). (b) Superposition of a monomer from the apo (white), holo (purple), and G3P‐bound (light blue) GAPN cryo‐EM models with the coenzyme and substrate shown. The N‐ and C‐termini, the oligomerization region with number of the three β‐strands, and the active site (dashed box) are delineated. (c) Blow‐up of the boxed region of (b) for the apo and holo GAPN models are compared. Arrows delineate the adenosine moiety, the nicotinamide moiety, C283, and the structural differences of residues 208–219. (d) Blow‐up of the boxed region of (b) for the apo and G3P‐bound models are compared with arrows delineating G3P and the structural differences of residues 208–219. (e) Comparison of subunits A of apo *S. pneumoniae* GAPN (white) with holo *T. tenax* GAPN (green, PDB accession 1UXP) and holo *T. tenax* GAPN with an effector (orange‐red, PDB accession 1UXU). The other three subunits of apo *S. pneumoniae* GAPN, subunits B‐D, are delineated with surface representations. The C‐terminal oligomeric regions of *S. pneumoniae* GAPN apo *T. tenax* GAPN with the AMP allosteric effector. (f) Blow up of just the C‐terminal regions of (e). (g) Cryo‐EM density of NADP^+^. (h) Cryo‐EM density of G3P.

We undertook cryo‐EM studies of *S. pneumoniae* GAPN to identify the underlying interactions with both coenzyme and substrate. Three high‐resolution structures were obtained that includes the apo form (Figure S1, Supporting Information; PDB accession 9DLB), the holo form (Figure S2; PDB accession 9DLA), and the G3P‐bound form (Figure S3; PDB accession 9DLC), with the statistics of these also reported here (Table S1). All GAPN structures elucidated here exhibit the conserved tetrameric architecture previously observed for other homologues (Figure 4a). These GAPN structures are also well superimposed with each other with only minor differences and both the coenzyme and G3P substrate are found within the active site (Figure 4b). Only subtle differences are incurred by binding either coenzyme or substrate, which is localized to the helix comprising residues 208–219. Specifically, residues 208–219 form a distorted helical structure in the apo form with large differences observed between apo and holo GAPN due to the direct interaction with the coenzyme's adenosine moiety (Figure 4c). Much smaller changes are observed between apo and G3P bound forms (Figure 4d). Similar rearrangements of residues 208–219 are also observed for *S. mutans* and *T. tenax* GAPN homologues upon coenzyme binding (Cobessi et al. 1999; Lorentzen et al. 2004; Pohl et al. 2002). Finally, the catalytic C283 residue is adjacent to both the nicotinamide ring of the coenzyme and the adjacent to the substrate (Figure 4c,d). This is expected for C283 to attack the substrate carbonyl and formation of a thiohemiacetal, which is followed by oxidation and transfer of electrons to NADP^+^ to form NADPH and hydrolysis of the thioester. The GAPN structures solved here by cryo‐EM are within 0.5–1 Å RMSD of the X‐ray crystal structures of *S. mutans* (Cobessi et al. 1999).

Structural studies such as those performed here identify the underlying reason as to why Gram‐positive GAPN enzymes are not allosterically regulated by effectors like that of *T. tenax* GAPN. Specifically, the C‐terminal region comprises part of the oligomerization region of the tetramer and is deeply buried (Figure 4e). However, in *T. tenax* GAPN, this C‐terminal region comprises the eight‐residue extension relative to all the Gram‐positive GAPN enzymes (Figure 2b, green), which is also the allosteric binding site for effectors (Figure 4f). Specifically, allosteric effectors, such as adenosine monophosphate (AMP) and glucose‐1‐phosphate (G1P), target this C‐terminus, inducing a conformational change that effects all subunits within the tetramer. This is shown here with *T. tenax* GAPN free (Figure 4f, green) and bound to AMP (Figure 4f, red). Thus, even if effectors could bind similarly to any of the Gram‐positive GAPN homologous, they could not incur changes to the C‐terminal region that is only present within *T. tenax* GAPN.

Structural studies do not shed light on the allosteric nature of G3P binding revealed by ITC binding experiments described within this study. Specifically, ITC indicates that G3P binding is negatively allosteric, considering that only one G3P substrate binds per GAPN tetramer, yet no significant changes are observed (Figure 4b). The structural changes that are observed for *S. pneumoniae* GAPN interactions are largely confined to residues 208–219, but this helix does not make direct contact with the other subunits (Figure 4e). Interestingly, compared to the coenzyme density (Figure 4g), the G3P density is much lower with little to no density observed for the phosphate group (Figure 4h). This likely reflects averaging over all monomers that include three subunits that do not engage G3P. The fact that ITC indicates that only one G3P is bound per tetramer and such changes are under the resolution of our cryo‐EM structural elucidations suggests that the underlying allosteric mechanism comprises only small perturbations. Alternatively, the allosteric coupling could be dynamic in nature and would therefore be difficult to identify through cryo‐EM.

### 2.4 Knockout of *S. pneumoniae* GAPN reduces infection

**FIGURE 5:**
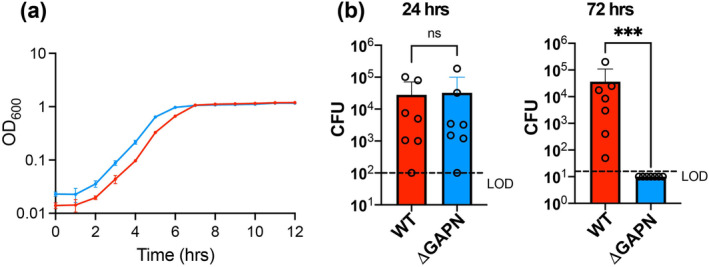
Comparison of growth and infection for *S. pneumoniae* WT versus GAPN knockout strains. (a) Growth curves for both *S. pneumoniae* WT (D39) and GAPN knockout (Δgapn) were monitored at the indicated time points at OD_600_ nm. (b) Burdens of *S. pneumoniae* WT and GAPN knockout strains detected as colony forming units (CFUs) from bronchoalveolar lavage fluid collected at 24 and 72 h post infection of C57BL/6J wild‐type mice. The average CUFs are shown as bars along with standard deviations indicated above each bar (wild‐type mice, red; GAPN knockout, blue), with the limit of detection delineated for each experiment (LOD, dashed lines).

To address the biological significance of *S. pneumoniae* GAPN in both growth and infection, we produced a knockout of GAPN (*Δgapn*). Bacterial growth curves were monitored at 37°C by optical density at 600 nm (OD_600_) (Figure 5a). Interestingly, *S. pneumoniae* GAPN knockout provides a slight advantage in cell culture for proliferation over the *S. pneumoniae* wild‐type (WT). Considering our discoveries presented in‐depth below that reveal GAPN knockout in *S. pneumoniae* leads to metabolic reprogramming, this may not be entirely surprising. Namely, GAPN knockout results in increased glycogen storage with a simultaneous enhanced fatty acid metabolism. Thus, energy production through fatty acid metabolism, that is, beta‐oxidation, provides an efficient means for cellular survival that results in a slight improvement to proliferation in cell culture.

We next sought to address how *S. pneumoniae* infection is altered in the absence of GAPN in vivo. Bronchoalveolar lavage fluid was collected from infected mice and plated on selective agar to monitor *S. pneumoniae* colony forming units (CFUs). No change in infection was observed for the first 24 h between *S. pneumoniae* WT and GAPN knockout strains. However, at 72 h there was a marked difference between these two strains that indicates GAPN knockout severely reduces *S. pneumoniae* infection (Figure 5b). Compared to *S. pneumoniae* WT, which was detected in the lungs of all mice with a median of 8.5 × 10^3^ CFUs at 72 h, GAPN knockout bacteria were completely cleared from the bronchoalveolar lavage fluid by this time point. Considering the underlying oxidative stress experienced by bacteria during infection of the host, this could be due to several factors that include the role of GAPN as a key regulator of cellular redox in recycling NADPH. Additionally, studies below indicate GAPN knockout leads to an increase in fatty acid metabolism. Considering that oxidative stress has been observed to reduce fatty acid metabolism (Doi et al. 2014), this likely leads to additional cell stress for the GAPN knockout strain.

### 2.5 Proteomic profiling of GAPN knockout reveals a switch to glycogen storage in cell culture

**FIGURE 6:**
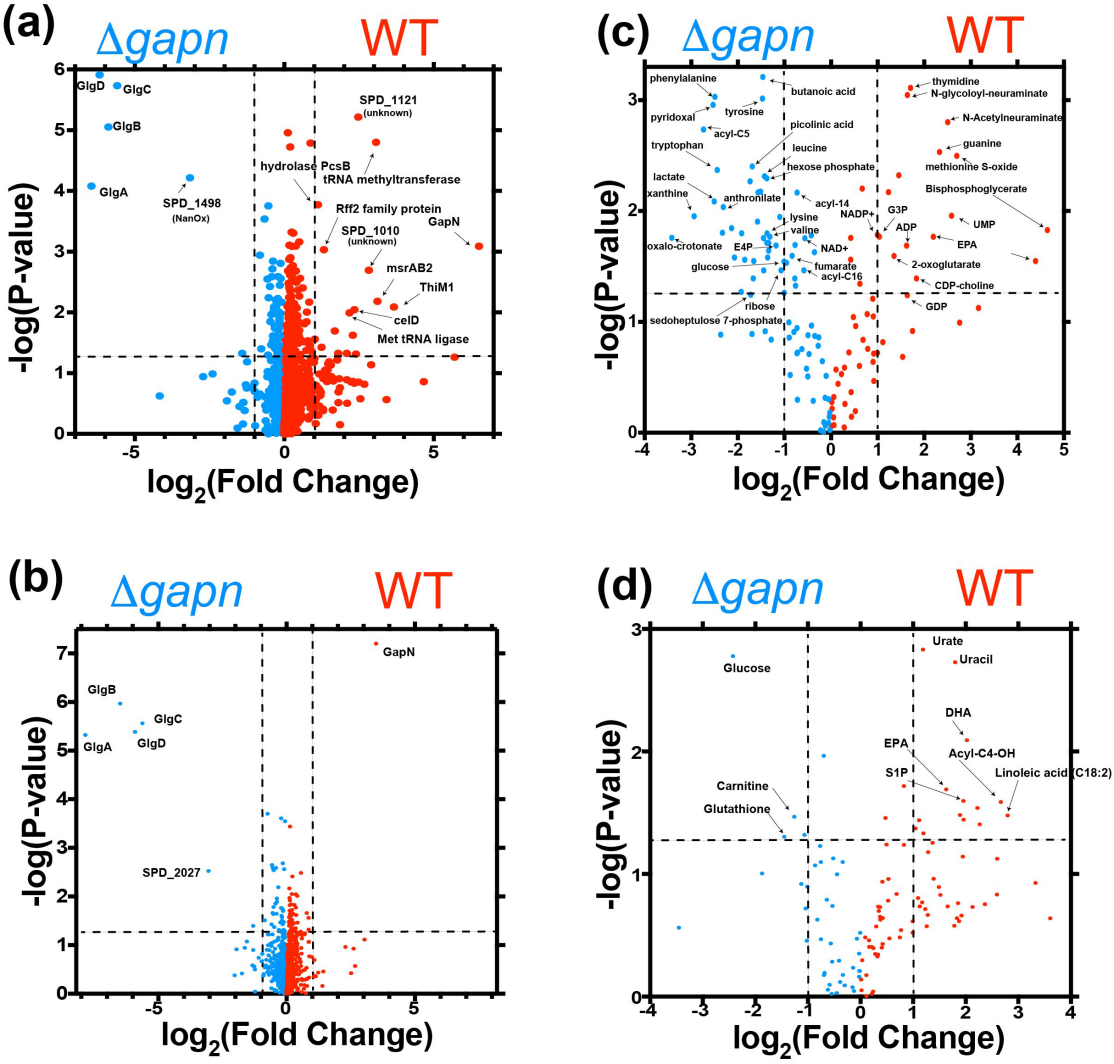
Proteomic and metabolic changes due to *S. pneumoniae* GAPN knockout. (a) Volcano plot indicating proteins that increase (blue, left) and decrease (red, right) upon GAPN knockout of cells grown in culture. (b) Volcano plot indicating proteins that increase (blue, left) and decrease (red, right) upon GAPN knockout for cells derived from infected mice. (c) Volcano plot indicating metabolites that increase (blue, left) and decrease (red, right) upon GAPN knockout of cells grown in culture. (d) Volcano plot indicating metabolites that increase (blue, left) and decrease (red, right) upon GAPN knockout derived from the BAL fluid of infected mice. Intensities were derived and compared in triplicate between WT and GAPN knockout strains.

To probe the underlying reasons as to why knockout of GAPN within *S. pneumoniae* leads to such a remarkable diminishment in virulence, we performed mass spectrometry to identify changes to the bacterial proteome (Figure 6a and Table S2). Five enzymes are upregulated upon GAPN knockout that include four enzymes directly involved in glycogenesis, GlgA, GlgB, GlgC, and GlgD, along with a putative oxidoreductase, SPD_1498, which likely also contributes to energy storage. Specifically, GlgC (glucose‐1‐phosphate adenylyltransferase) that often functions with the regulatory subunit GlgD activates glucose by producing ADP‐glucose to extend the glycogen chain through GlgA (glycogen synthase). GlgB (1,4‐alpha‐glucan branching enzyme) modifies the glycogen linkages. Thus, knockout of GAPN results in metabolic rewiring for *S. pneumoniae* glycogen storage.

Interestingly, the only other protein that is significantly upregulated is SPD_1498, which shares high sequence similarity to the sialic acid scavenging enzyme N‐acetylneuraminate lyase/oxidoreductase (NanOx). NanOx is responsible for the reversible oxidation–reduction of 2,7‐anhydro‐N‐acetylneuraminic acid (2,7‐anhydro‐Neu5Ac) to N‐acetylneuraminic acid (Neu5Ac) and simultaneously uses NAD^+^/NADH (Bell et al. 2020). NanOx activity could be used to help regulate the redox potential of the cell in the absence of GAPN and simultaneously feed sialic acid into the same metabolic pathway as that of glucosamine salvage. Glucosamine salvage ultimately feeds into NagB, which produces fructose‐6‐phosphate for glycolysis or can be used for glycogenesis. Potentially of wider interest is our discovery here that sialic acid is present within the media used to grow *S. pneumoniae* (Table S3). Combined with the identification below of sialic acid usage in the *S. pneumoniae* WT strain, NanOx may be upregulated upon a loss of such metabolites described further in the next section. Collectively, these findings indicate that glycolysis is dramatically disrupted upon GAPN knockout and *S. pneumoniae* is rewired for glycogen storage instead of glucose metabolism.

In contrast to the identified enzymes that increase upon GAPN knockout, there are several proteins that are decreased relative to the WT strain. Several of these have unknown functions with little sequence comparison to known proteins. However, there are changes to enzymes involved in redox regulation, tRNA biology, and glucose metabolism. These include msrAB2 that reduces thiols, along with two tRNA modifying enzymes downregulated that include a tRNA methyltransferase and tRNA ligase. Finally, a component of the phosphotransferase system responsible for cellobiose uptake is also diminished in GAPN knockout, called celD (Paixão et al. 2015). Considering cellobiose is a disaccharide of glucose that is normally metabolized to glucose, such findings once again indicate a disruption in glucose metabolism.

Proteomic comparisons were also conducted between cells grown after infection for *S. pneumoniae* WT and GAPN knockout strains (Figure 6b and Table S4). While no bacteria were detected in bronchoalveolar lavage fluid for the GAPN knockout by 72 h post‐infection, a small number of colonies were detected in the lung tissue homogenates. *Streptococcus pneumonia* WT and GAPN knockout cells from lung tissue were grown in triplicate until saturation. Similar to the differences identified in cell culture, *S. pneumoniae* cells post‐infection also exhibit an increase in glycogen storage enzymes post infection for the GAPN knockout strain, including GlgA, GlgB, GlgC, and GlgD. However, as opposed to SPD_1498, potentially a NanOx enzyme responsible for sialic acid metabolism, SPD_2027 is now increased within the GAPN knockout strain relative to the WT strain. SPD_2027 is listed as a thiamine binding protein that remains a domain of unknown function. However, its structure was solved nearly 20 years ago by the structural genomics consortium (PDB accession 2IBO), which reveals a tetramer. Using 2IBO for a Dali structural comparison (Holm and Sander 1993), there is high homology of SPD_2027 to transcription factors, and thus, the function of this protein remains unclear. Nonetheless, the largest differences both prior and post infection for the GAPN knockout strain primarily indicate a switch to glycogen storage rather than sugar metabolism.

### 2.6 Metabolic profiling of GAPN knockout reveals a switch to lipid storage and key antimicrobial metabolites in cell culture

Mass spectrometry was also used to identify the metabolic differences upon *S. pneumoniae* GAPN knockout, which reveals a switch to alternative energy pathways likely due to reduced sugar metabolism (Figure 6c and Table S5). For example, GAPN knockout increases acyl‐carnitine chains, such as acyl‐C5 and acyl‐C14, suggesting an increase in lipid metabolism to offset the decrease in sugar metabolism. Simultaneously, specific lipids, such as eicosapentaenoic acid (EPA), are diminished within the GAPN knockout strain that is consistent with the rewiring lipid metabolism for energy production. In addition to the observed increases in lipids, there are concurrent increases in numerous amino acids that are observed upon GAPN knockout, suggesting proteins are also being catabolized for energy. Increases in tryptophan along with its catabolic metabolites, picolinic acid and oxalocrotonate, and increases in tyrosine along with its catabolite fumarate support the catabolism of amino acids for energy. This increase in lipid and protein metabolism, GAPN knockout also leads to the production of metabolytes that would further hamper infection. For example, in the *S. pneumoniae* GAPN knockout strain there is an increase in lactate and butanoic acid, also known as butyrate, that indicates an increase in fermentation. The increase in butyrate upon GAPN knockout is a particularly interesting finding. Butyrate is a key modulator of antimicrobial activity, which modulates bacterial signaling but is also known to modulate host gene expression through its inhibition of histone deacetylase (Du et al. 2021). Butyrate also induces the antimicrobial peptide, LL‐37 (Schauber et al. 2003), which would be expected to further diminish virulence of the GAPN knockout strain consistent with its reduced infection.

GAPN knockout also affects the PPP, which is significantly increased in this mutant strain. For example, in *S. pneumoniae* GAPN knockout strain there is a reduction in glycolytic intermediates that includes G3P, the substrate for both GAPN and GAPDH, along with 1/2‐3‐bisphosphoglycerate, the product of GAPDH, which indicates a metabolic shift away from glycolysis due to the removal of GAPN. There may even be a more general shift away from sugar metabolism, as sialic acid intermediates derived from the media, such as N‐Acetylneuraminate and N‐glycolyl‐neuraminate, are higher in the WT strain. Evidence for GAPN knockout resulting in a shift to the PPP is further indicated by an increase in PPP intermediates that include glucose 6‐phosphate—substrate for the entry step of the oxidative phase, erythrose‐4‐phosphate (E4P), and sedoheptulose 7‐phosphate—non‐oxidative phase metabolites. This suggests compensatory activation of the PPP to generate NADPH upon loss of GAPN‐derived NADPH generation. Moreover, NADP^+^ is higher in the WT strain, not the GAPN knockout strain where it would be expected to accumulate. However, it is important to note that our methods cannot detect NADPH due to ionization that makes it difficult to monitor such changes. GAPN knockout also results in a simultaneous diminishment in nucleotide production, observed by reduced ADP, GMP, thymidine, and guanine relative to the WT strain. Although NADPH is usually undetectable due its instability, these findings suggest that a metabolic switch to the PPP is a regulatory response to redox changes to produce more NADPH via an alternative route due to the loss of GAPN. Notably, GAPN knockout was associated with extensive reprogramming of aromatic amino acid metabolism (phenylalanine, tyrosine, tryptophan), with elevation in tryptophan metabolites anthranilate and picolinic acid in the kynurenine/NAD^+^ synthesis pathway.

A metabolic comparison of the bronchoalveolar lavage fluid was also performed between *S. pneumoniae* GAPN knockout and WT strains (Figure 6d and Table S6). Although both the pathogen and the host contribute to the metabolic environment, there are observed differences consistent with both the proteomic and metabolic differences observed in cell culture. Specifically, several metabolites are higher in the GAPN knockout infected mice that include glucose, glutathione, and carnitine. The increase in glucose is consistent with the reduced ability of this mutant strain to metabolize glucose relative to that of mice infected with the WT strain. The increase in glutathione may be consistent with increased oxidative stress in the GAPN knockout strain due to a decrease in NADPH production. Conversely, it may be produced by the host and simply utilized by the *S. pneumoniae* WT strain during infection, while therefore present at elevated levels during infection of the *S. pneumoniae* GAPN knockout strain that is already cleared. As for the observed increase in carnitine, this is consistent with the need for the GAPN knockout strain to utilize lipid metabolism. Carnitine is conjugated to lipids during lipid metabolism, which we show is insufficient for bacterial survival during infection and likely results in bacterial cell lysis. Furthermore, stress has been shown to diminish carnitine uptake in other Gram‐positive bacteria (Schuster et al. 2016), which may also account for excess levels in the bronchoalveolar lavage fluid of mice infected with the GAPN knockout strain. Conversely, several metabolites are diminished in the GAPN knockout infected mice. For example, sphingosine‐1‐phosphate (S1P) is decreased, which could be due to disruption of NADPH concentrations that are necessary for sphingolipid biosynthesis. S1P is a marker of hypoxia (Sun et al. 2016), suggesting that genetic ablation of GAPN mitigates the hypoxic phenotype in BALF in vivo. Interestingly, two highly unsaturated omega‐3 fatty acids, EPA and docosahexaenoic acid (DHA), are increased in GAPN knockout infected mice. These observations may be consistent with the rewiring of lipid metabolism within the GAPN knockout strain perhaps as a function of increased elongation and desaturation, a process involved in glycolytic NAD recycling (Kim et al. 2019). Additional contributions may come from the host, as both EPA and DHA are antimicrobial (Ribeiro‐Vidal et al. 2020). For example, decreases in the levels of urate in the BALF upon infection with *S. pneumoniae* GAPN knockout is consistent with a preservation of ATP pool and a decrease in the breakdown and deamination to hypoxanthine, xanthine, and urate—a hallmark of (ischemic, hemorrhagic, and septic) hypoxia in the lung (Liu et al. 2022). Thus, their production may simply be due to the increased virulence of the WT strain relative to that of the GAPN strain that is cleared more efficiently. In general, these in vitro and in vivo studies indicate a rewiring of metabolism due to GAPN knockout and a shift to lipid metabolism that sensitizes the pathogen to host clearance.

## 3 DISCUSSION

### 3.1 *Streptococcus pneumoniae* GAPN exhibits structural similarities to other homologues but may be unique in its biochemical regulation

Our studies here have been the first to characterize *S. pneumoniae* GAPN, revealing both similarities and potentially important differences to other homologues. For example, cryo‐EM structural studies here of *S. pneumoniae* apo GAPN, coenzyme‐bound GAPN, and substrate‐bound GAPN reveal a highly conserved enzyme with similar conformational changes between forms to previous homologues probed by X‐ray crystallography (Cobessi et al. 1999; Lorentzen et al. 2004; Pohl et al. 2002). Even the relatively slow *k*_cat_ of 14 s^−1^ for *S. pneumoniae* GAPN is consistent with other GAPN homologues that range from ~6 to 60 s^−1^ (Eisenberg et al. 2022; Lorentzen et al. 2004; Marchal and Branlant 2002). However, we have also discovered that G3P substrate binding is negatively allosteric with one substrate molecule binding to each monomer within the *S. pneumoniae* GAPN tetramer. This may be unique to *S. pneumoniae* GAPN, as X‐ray crystal structures of G3P bound to other homologues do exhibit the expected substrate density. In contrast, we observe only weak density of G3P within each of subunits within the cryo‐EM structure of *S. pneumoniae* GAPN due to averaging among the four subunits. This could suggest that the induced conformational changes between subunits is below the resolution of our cryo‐EM single particle reconstructions or may be dynamic in nature. Despite our current limitations in being able to identify the underlying mechanism of negative cooperativity, these studies identify a sophisticated mechanism of metabolic flux. For example, negative cooperativity for *S. pneumoniae* GAPN reveals this enzyme must limit its catalytic function and may simply use oligomerization as a means for stability.

Finally, the large difference between the *K*_*D*_ and *K*_*M*_ for GAPN and its G3P substrate also provides biochemical insight into the potential rate‐limiting step. Specifically, the slow turnover monitored for GAPN (*k*_cat_ = 14 s^−1^) allows an estimation of the off‐rate of the G3P substrate that is also very slow (*k*_off_ = 0.0084 s^−1^). Unsurprisingly, this indicates that the 3‐PG product binds more weakly than the G3P substrate but even if this is 100‐fold weaker it also suggests that product release may be rate‐limiting. This is indeed the case for GAPDH (Canellas and Cleland 1991), as well as other oxidoreductases (Rozovsky et al. 2001).

### 3.2 Metabolic rewiring due to GAPN knockout reduces infection

We have discovered that knockout of *S. pneumoniae* GAPN induces significant metabolic reprogramming that collectively diminishes the ability to sustain infection. The loss of *S. pneumoniae* GAPN was expected to disrupt NADPH production critical for maintaining redox but was found to also create a bottleneck in glycolysis that forces metabolism towards alternative pathways. Specifically, the GAPN knockout strain exhibits a marked increase in glycogen storage, as evidenced by the upregulation of glycogen synthesis enzymes, GlgA, GlgB, GlgC, and GlgD. This increase in glycogen storage enzymes is observed in GAPN knockout cells grown in vitro but also for cells collected post infection from the bronchoalveolar lavage fluid of mice. Thus, the metabolic shift suggests that the bacteria are prioritizing energy storage from sugars over the immediate utilization of these sugars through glycolysis, potentially as a survival strategy under the stress of reduced glycolytic activity. Concurrently, there is a pronounced increase in fatty acid metabolism, with elevated levels of acyl‐carnitine chains indicating enhanced reliance on beta‐oxidation as an alternative for energy production. While this shift to lipid metabolism is viable in cell culture, it makes the bacteria particularly sensitive to oxidative stress during infection (Doi et al. 2014). Furthermore, GAPN knockout results in the production of metabolites that increase the host's innate immune response, such as butyrate production that also drives antimicrobial activities (Du et al. 2021; Schauber et al. 2003). In fact, short chain fatty acids, like butyrate, have shown very recent promise in inhibiting *S. pneumoniae* infections (Lim et al. 2024). These combined effects of impaired glycolysis, increased oxidative stress sensitivity due to a shift in lipid metabolism, and an increased host innate immune response leads to a reduced virulence within 72 h of infection. This metabolic rewiring underscores the critical role of GAPN in maintaining *S. pneumoniae* metabolic flexibility and resilience during infection.

### 3.3 Implications for pneumococcal disease

The findings presented here provide a comprehensive view of how GAPN influences the metabolism and virulence of *S. pneumoniae*. The discovery of negative cooperativity in G3P binding adds a new layer of complexity to our understanding of GAPN's regulatory mechanisms. The metabolic reprogramming observed in the GAPN knockout strain underscores this enzyme's critical role in maintaining metabolic balance, with disruptions leading to increased oxidative stress and reduced bacterial virulence. The remarkable shift in metabolism upon knockout of GAPN indicates a much more important role in *S. pneumoniae* metabolism than may have been previously recognized. This could be due to the versatile role of *S. pneumoniae* GAPDH that may have adapted to perform a much more diverse set of functions with a subsequent reliance on GAPN for glycolytic flux (Seidler and Seidler 2013). Studies here suggest that GAPN could be a valuable target for therapeutic intervention, offering new avenues for the treatment of pneumococcal infections. Either targeting GAPN or treating the host with molecules shown here to increase upon GAPN knockout, such as butyrate, could add to novel treatments currently under investigation (Cools et al. 2021).

## 4 CONCLUSIONS

This study provides the first comprehensive characterization of *S. pneumoniae* GAPN, revealing its critical role in metabolic balance and infection capability. Through a combination of structural, biochemical, and biological assays, we demonstrated that GAPN is negatively allosteric with respect to its G3P substrate and that its knockout leads to significant metabolic reprogramming. This reprogramming includes an increase in glycogen storage and enhanced fatty acid metabolism, which compromises the ability of *S. pneumoniae* to manage oxidative stress and sustain infection. These findings highlight GAPN as a potential target for therapeutic intervention in pneumococcal diseases, as these studies identify GAPN as far more critical to *S. pneumoniae* infection than previously thought.

## 5 MATERIALS AND METHODS

### 5.1 Cloning and recombinant purification of *S. pneumoniae* GAPN

The *S. pneumoniae gapn* gene (UniProt accession number A0A0H2ZNN4_STRP2) was PCR amplified from genomic DNA from *S. pneumoniae* strain D39 and inserted with an N‐terminal 6xHis tag into NdeI‐cleaved pET21b using overlapping primers. The enzyme was expressed in BL21(DE3) cells in Luria Broth at 25°C for 5 h. Cell pellets from a typical 2 L growth were lysed via sonication in “Ni Buffer A” (50 mM KPO4, pH 7.5, 100 mM KCl, 10 mM imidazole), applied to a 20 mL Ni‐affinity column (Sigma), and eluted in “Ni Buffer B” (Ni Buffer A plus 1M imidazole). Eluted fractions were concentrated and applied to a Superdex‐200 (Cytiva) in “Final Buffer” (50 mM HEPES, pH 7.5, 50 mM NaCl, 2 mM DTT). Fractions comprising GAPN were concentrated and frozen at −80°C until further use.

### 5.2 Biochemical studies

UV‐kinetic studies were monitored on a SpectraMax 384 Plus (Molecular Devices, Inc.) using a 1 mm cuvette at 25°C. Reaction volumes comprised 300 μL total in “Final Buffer” and were monitored for 1 min using 40 nM GAPN using the indicated concentrations of G3P (Caymen Chemical Company, Ann Arbor, MI), NADP^+^ (MP Biomedicals, LLC, Solon, OH), and E4P (Sigma). Data were fit to the Michaelis–Menton equation using GraphPad Prism version 4.0 (GraphPad Software Inc., San Diego, CA).

Isothermal titration calorimetry (ITC) was performed on a MicroCal VP‐ITC with well samples containing 100 μM GAPN and the indicated additions of G3P and NADP^+^. “Final Buffer” was used for all ITC experiments. Presented data represent averages and uncertainties performed in triplicate at 25°C and processed using Origin software provided with the MicroCal VP‐ITC.

### 5.3 Cryo‐EM structural studies

For the *S. pneumoniae* GAPN, purified protein was concentrated to 90 μM (~4 mg mL^−1^) in “Final Buffer” and used alone for free GAPN or with either 1 mM added G3P or 1 mM NADP^+^. For grid preparations, 3 μL of ~3 mg mL^−1^ sample was applied to plasma‐cleaned C‐flat holy carbon grids (1.2/1.3, 400 mesh) and frozen using a Vitrobot Mark IV (Thermo Fisher Scientific), with the environmental chamber set at 100% humidity and 4°C. The grids were blotted for 3.0 s and then flash frozen in liquid‐nitrogen‐cooled liquid ethane. A full description of the cryo‐EM data collection can be found in Table S1. All three data sets of GAPN were collected on a Krios microscope located at the Pacific Northwest Center for Cryo‐EM. Data for cryo‐EM 3D single particle reconstructions were processed in cryoSPARC (Punjani et al. 2017) where vovies were motion corrected using MotionCor2 (Iverson et al. 2020) and their contrast transfer functions estimated using Gctf (Zhang 2016). Class averages were selected and used for template picking. The particles were extracted with a box size of 336 × 336 pixels and subjected to 2D classification. Classes with clear visible secondary features were selected and subjected to ab initio reconstruction, 3D classification, and refinement. The resolution of the final maps was based on the gold‐standard Fourier shell correlation (FSC) measurement. Initial models for both substrate‐free and the substrate‐bound complex were built in Chimera (Pettersen et al. 2004), which used the previously determined structure of the *S. mutans* GAPN as an initial model (Cobessi et al. 1999; Cobessi et al. 2000). Models were then refined in Coot (Emsley et al. 2010) and Phenix (Adams et al. 2010).

### 5.4 GAPN knockout, monitoring growth, and infection

*Streptococcus pneumoniae* was grown in Todd Hewitt Broth with 5% Yeast Extract (BD Bacto™) at 37°C with 5% CO_2_. Agar plates were comprised of Tryptic Soy Broth (MP Biomedicals) supplemented with 5 μg mL^−1^ neomycin and 5000 units/plate fresh catalase (Worthington Biomedical Corporation) and incubated at 37°C with 5% CO_2_ for 18 h for CFU enumeration. *Streptococcus pneumoniae* serotype 2 strain D39 was used as the WT strain for all studies. The genetic knockout of GAPN was performed in this strain using standard methods (Echlin and Rosch 2020). Specifically, 500 base pair overhangs along with an internal sequence encoding for kanamycin from the Janus cassette was purchased from Elegen (San Carlos, CA) and linear DNA was used to transform bacteria in minimal media with competence stimulating peptides (Davis et al. 2008).

Male and female C57BL/6J wild‐type mice were purchased from The Jackson Laboratory (RRID:IMSR_JAX000664). Mice were infected at 6–8 weeks of age. Infections were performed intratracheally with inhaled isoflurane anesthesia. Mice were infected with 10^7^ CFU/mouse and sacrificed either 24 or 72 h post infection for the collection of bronchoalveolar lavage fluid and lung tissue. Lavages were collected in 1 mL PBS from cannulated trachea. Lung tissue was homogenized in PBS using a Bullet Blender homogenizer (Stellar Scientific, Baltimore, MD) and were centrifuged for 30 s at 500*g* followed by plating serial dilutions of the supernatants on selective agar media.

### 5.5 Study approval

Studies in mice were approved by the Animal Care and Use Committee, protocol 00927, of the University of Colorado School of Medicine. All work with live *S. pneumoniae* was conducted in BSL2 facilities and approved by the Institutional Biosafety committee, protocol 1418, of the University of Colorado School of Medicine.

### 5.6 Proteomic comparisons between *S. pneumoniae* WT and GAPN knockout

*Streptococcus pneumoniae* WT and GAPN knockout cells were grown in triplicate in 10 mL for 12 h and centrifuged for mass spectrometry analysis. These included *S. pneumoniae* WT and GAPN knockout strains prior to infection and colonies derived from the infected mice post‐infection. For analysis, samples were reduced, alkylated, and digested using S‐Trap™ micro filters (Protifi, Huntington, NY) according to the manufacturer's protocol. Digested peptides were cleaned using Pierce™ C18 Spin Tips (Thermo Scientific) according to the manufacturer's protocol, dried in a vacuum centrifuge, and resuspended in 0.1% formic acid in mass spectrometry‐grade water. Peptides were loaded into autosampler vials and analyzed directly using a NanoElute liquid chromatography system (Bruker, Germany) coupled with a timsTOF SCP mass spectrometer (Bruker, Germany). Peptides were separated on a 75 μm i.d. × 15 cm separation column packed with 1.9 μm C18 beads (Bruker, Germany) over a 90‐min elution gradient. Buffer A was 0.1% FA in water and buffer B was 0.1% FA in acetonitrile. Instrument control and data acquisition were performed using Compass Hystar (version 6.0) with the timsTOF SCP operating in parallel accumulation‐serial fragmentation (PASEF) mode under the following settings: mass range 100–1700 m/z, 1/k/0 Start 0.7 End 1.3 V s^−1^ cm^−2^; ramp accumulation times were 166 ms; capillary voltage was 4500 V, dry gas 8.0 L min^−1^, and dry temp 200°C. The PASEF settings were 5 MS/MS scans (total cycle time, 1.03 s); charge range 0–5; active exclusion for 0.2 min; scheduling target intensity 20,000; intensity threshold 500; collision‐induced dissociation energy 10 eV.

Fragmentation spectra were searched against the UniProt *S. pneumoniae* D39 proteome database (ID: UP000001452) using the MSFragger‐based FragPipe computational platform (Kong et al. 2017). Contaminants and reverse decoys were added to the database automatically. The precursor‐ion mass tolerance and fragment‐ion mass tolerance were set to 15 and 20 ppm, respectively. Fixed modifications were set as carbamidomethyl (C), and variable modifications were set as oxidation (M), two missed tryptic cleavages were allowed, and the protein‐level false discovery rate (FDR) was ≤1%.

### 5.7 Metabolic comparisons between *S. pneumoniae* WT and GAPN knockout

Metabolic comparisons were made between *S. pneumoniae* WT and GAPN knockout cells as described for proteomic analysis along with comparisons of the bronchoalveolar lavage fluid drawn from infected mice. Metabolites were extracted from the *S. pneumoniae* cells through the addition of lysis buffer (50:30:20 (v/v/v) methanol:acetonitrile:water) at a ratio of 1 × 10^8^ cells mL^−1^ and the samples were vortexed at 4°C for 30 min. Samples were then centrifuged (18,000 rcf, 10 min, 4°C) and the supernatant removed to autosampler vials for untargeted metabolomic analysis by UHPLC–MS analysis (Thermo Vanquish UHPLC/Thermo Orbitrap Exploris 120 [Thermo Fisher Scientific, Waltham, MA]). Bronchoalveolar lavage fluid was extracted in a similar procedure except metabolites were extracted at a ratio of 1:10 with lysis buffer. Each sample was analyzed in both positive and negative mode using duplicate, separate runs utilizing a 5 min method (Nemkov et al. 2019). Mass spectra features in the data were identified, annotated and quantified (by peak area) using El‐Maven v0.12.0 (Agrawal et al. 2019), which were collated into a global metabolomics analysis. MetaboAnalyst 5.0 (Pang et al. 2021) was used for statistical analyses (PA, Heatmap) and pair‐wise comparisons.

## AUTHOR CONTRIBUTIONS

**Eunjeong Lee:** Conceptualization; methodology; investigation; data curation. **Anthony Saviola:** Investigation; data curation. **Shaun Bevers:** Investigation. **Jasmina S. Redzic:** Conceptualization; investigation; data curation. **Sean P. Maroney:** Investigation. **Steven Shaw:** Investigation. **Emily Tamkin:** Investigation. **Sam Fulte:** Investigation. **Travis Nemkov:** Conceptualization. **Nancy Meyer:** Investigation. **Angelo D'Alessandro:** Investigation; conceptualization. **Kirk C. Hansen:** Conceptualization; investigation. **Sarah E. Clark:** Conceptualization; investigation. **Elan Eisenmesser:** Conceptualization; investigation; funding acquisition; writing – original draft; writing – review and editing; methodology; formal analysis; data curation; supervision.

## Supporting information


**Figure S1.** Workflow for cryo‐EM data processing for apo GapN. (a) Raw representative cryo‐EM micrographs. (b) Representative 2D class averages of apo GapN. (c) Workflow of cryo‐EM data processing for the apo GapN. (d) Angular distribution heat map of particle projections for the apo GapN. (e) Gold standard Fourier shell correlation (FSC) curve for apo GapN reconstructions. Solid line represents the overall nominal resolution of each reconstruction at 0.143 FSC calculated by CryoSPARC.
**Figure S2**. Workflow for cryo‐EM data processing for holo GapN with NADP^+^. (a) Workflow of cryo‐EM data processing for the holo GapN. (b) Representative 2D class averages of picked particles collected from holo GapN. (c) Angular distribution heat map of particle projections for the holo GapN. (e) Gold standard Fourier shell correlation (FSC) curve for holo GapN reconstructions. Solid line represents the overall nominal resolution of each reconstruction at 0.143 FSC calculated by CryoSPARC.
**Figure S3**. Workflow for cryo‐EM data processing for G3P bound GapN. (a) Workflow of cryo‐EM data processing for the substrate bound GapN. (b) Representative 2D class averages of picked particles collected from substrate bound GapN. (c) Angular distribution heat map of particle projections for the G3P bound GapN. (e) Gold standard Fourier shell correlation (FSC) curve for G3P bound GapN reconstructions. Solid line represents the overall nominal resolution of each reconstruction at 0.143 FSC calculated by CryoSPARC.
**Table S1**. Data collection and model refinement of Apo GAPN, the GAPN/NADP+ complex, and the GAPN/G3P complex.

**Table S2.** Proteomic comparison of GAPN knockout and WT *S. pneumoiae* strain D39 grown in culture.
**Table S3**. Metabolic analysis of media used to grow *S. pneumoniae*.
**Table S4**. Proteomic comparison of GAPN knockout and WT *S. pneumoniae* strain D39 grown in culture post infection.
**Table S5**. Metabolic analysis comparison of GAPN knockout and WT *S. pneumoniae* strain D39 grown in culture post infection.
**Table S6**. Metabolic analysis comparison of GAPN knockout and WT *S. pneumoniae* within the BAL fluid of infected mice.
