# Supplementary material for: Streptococcus pneumoniae GAPN is a key metabolic player necessary for host infection
Source: Protein Sci. 2024 Dec 11;34(1):e5253. doi: 10.1002/pro.5253 (PMC11633051; doi:10.1002/pro.5253)
Supplement: Supplementary file 1 — Figure S1. Workflow for cryo‐EM data processing for apo GapN. (a) Raw representative cryo‐EM micrographs. (b) Representative 2D class averages of apo GapN. (c) Workflow of cryo‐EM data processing for the apo GapN. (d) Angular distribution heat map of particle projections for the apo GapN. (e) Gold standard Fourier shell correlation (FSC) curve for apo GapN reconstructions. Solid line represents the overall nominal resolution of each reconstruction at 0.143 FSC calculated by CryoSPARC. Figure S2. Workflow for cryo‐EM data processing for holo GapN with NADP+. (a) Workflow of cryo‐EM data processing for the holo GapN. (b) Representative 2D class averages of picked particles collected from holo GapN. (c) Angular distribution heat map of particle projections for the holo GapN. (e) Gold standard Fourier shell correlation (FSC) curve for holo GapN reconstructions. Solid line represents the overall nominal resolution of each reconstruction at 0.143 FSC calculated by CryoSPARC. Figure S3. Workflow for cryo‐EM data processing for G3P bound GapN. (a) Workflow of cryo‐EM data processing for the substrate bound GapN. (b) Representative 2D class averages of picked particles collected from substrate bound GapN. (c) Angular distribution heat map of particle projections for the G3P bound GapN. (e) Gold standard Fourier shell correlation (FSC) curve for G3P bound GapN reconstructions. Solid line represents the overall nominal resolution of each reconstruction at 0.143 FSC calculated by CryoSPARC. Table S1. Data collection and model refinement of Apo GAPN, the GAPN/NADP+ complex, and the GAPN/G3P complex. [file PRO-34-e5253-s002.docx]

**SUPPLEMENTARY MATERIAL**

**Title: *Streptococcus pneumoniae* GAPN is a key metabolic player necessary for host infection.**

Eunjeong Lee^1^, Anthony Saviola^1^, Shaun Bevers^1^, Jasmina S. Redzic^1^, Sean P. Maroney^1^, Steven Shaw^2^, Emily Tampkin^2^, Sam Fulte^2^, Travis Nemkov^1^, Nancy Meyer^3^, Angelo D’Alessandro^1^, Kirk C. Hansen^1^, Sarah E. Clark^2^, and Elan Eisenmesser^1^.

^1^Department of Biochemistry and Molecular Genetics, School of Medicine, University of Colorado Anschutz Medical Campus, School of Medicine, Aurora, CO 80045

^2^Department of Otolaryngology, School of Medicine, University of Colorado Anschutz Medical Campus, School of Medicine, Aurora, CO 80045

^3^Pacific Northwest Cryo-EM Center, Oregon Health and Science University, Portland, OR 97201

^†^Corresponding author: Elan.Eisenmesser@ucdenver.edu

Contents:

Figure S1

Figure S2

Figure S3

Table S1


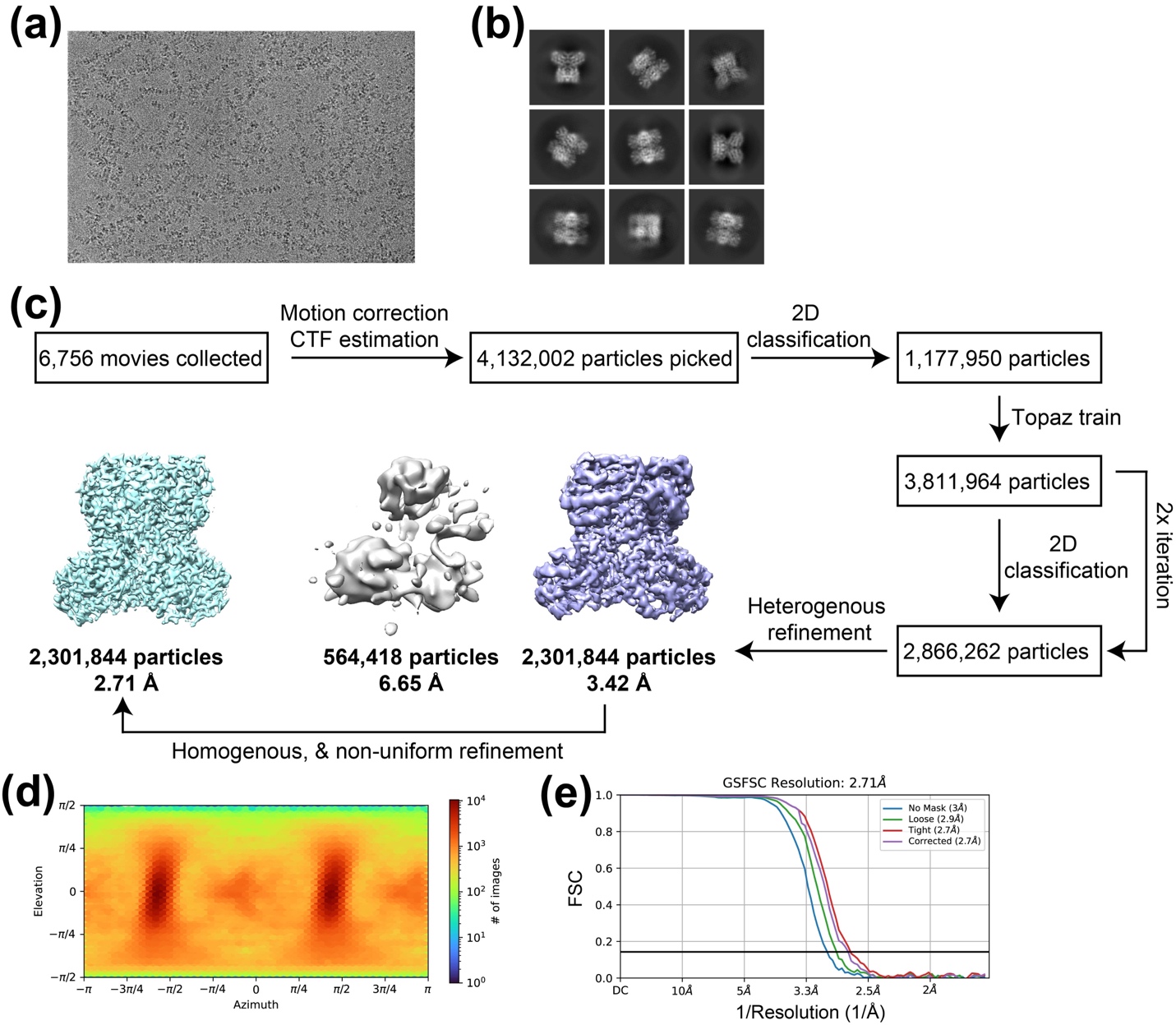


Figure S1. Workflow for cryo-EM data processing for apo GapN. (a) Raw representative cryo-EM micrographs. (b) Representative 2D class averages of apo GapN. (c) Workflow of cryo-EM data processing for the apo GapN. (d) Angular distribution heat map of particle projections for the apo GapN. (e) Gold standard Fourier shell correlation (FSC) curve for apo GapN reconstructions. Solid line represents the overall nominal resolution of each reconstruction at 0.143 FSC calculated by CryoSPARC.


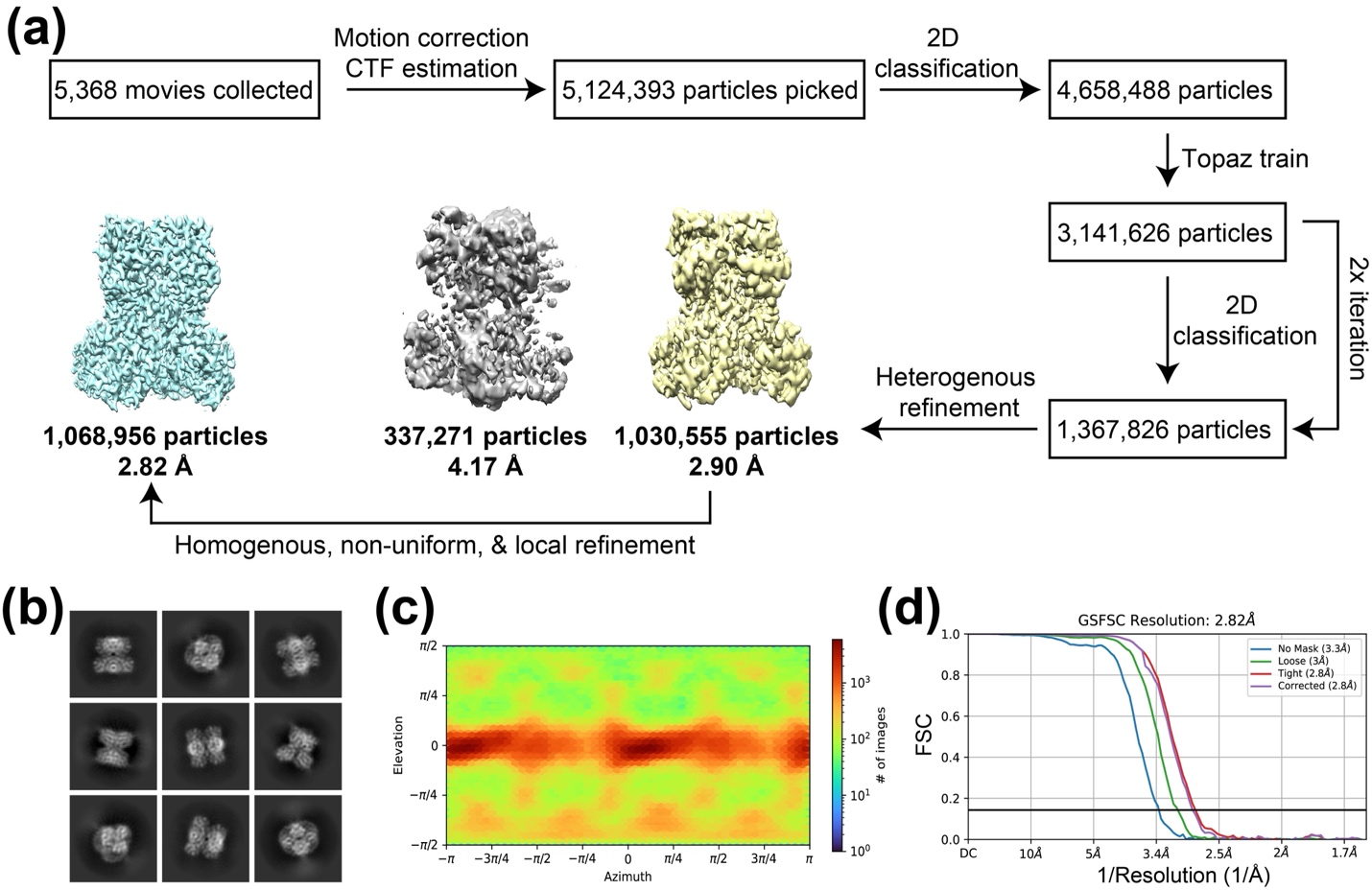


Figure S2. Workflow for cryo-EM data processing for holo GapN with NADP^+^. A) Workflow of cryo-EM data processing for the holo GapN. B) Representative 2D class averages of picked particles collected from holo GapN. C)Angular distribution heat map of particle projections for the holo GapN. E) Gold standard Fourier shell correlation (FSC) curve for holo GapN reconstructions. Solid line represents the overall nominal resolution of each reconstruction at 0.143 FSC calculated by CryoSPARC.


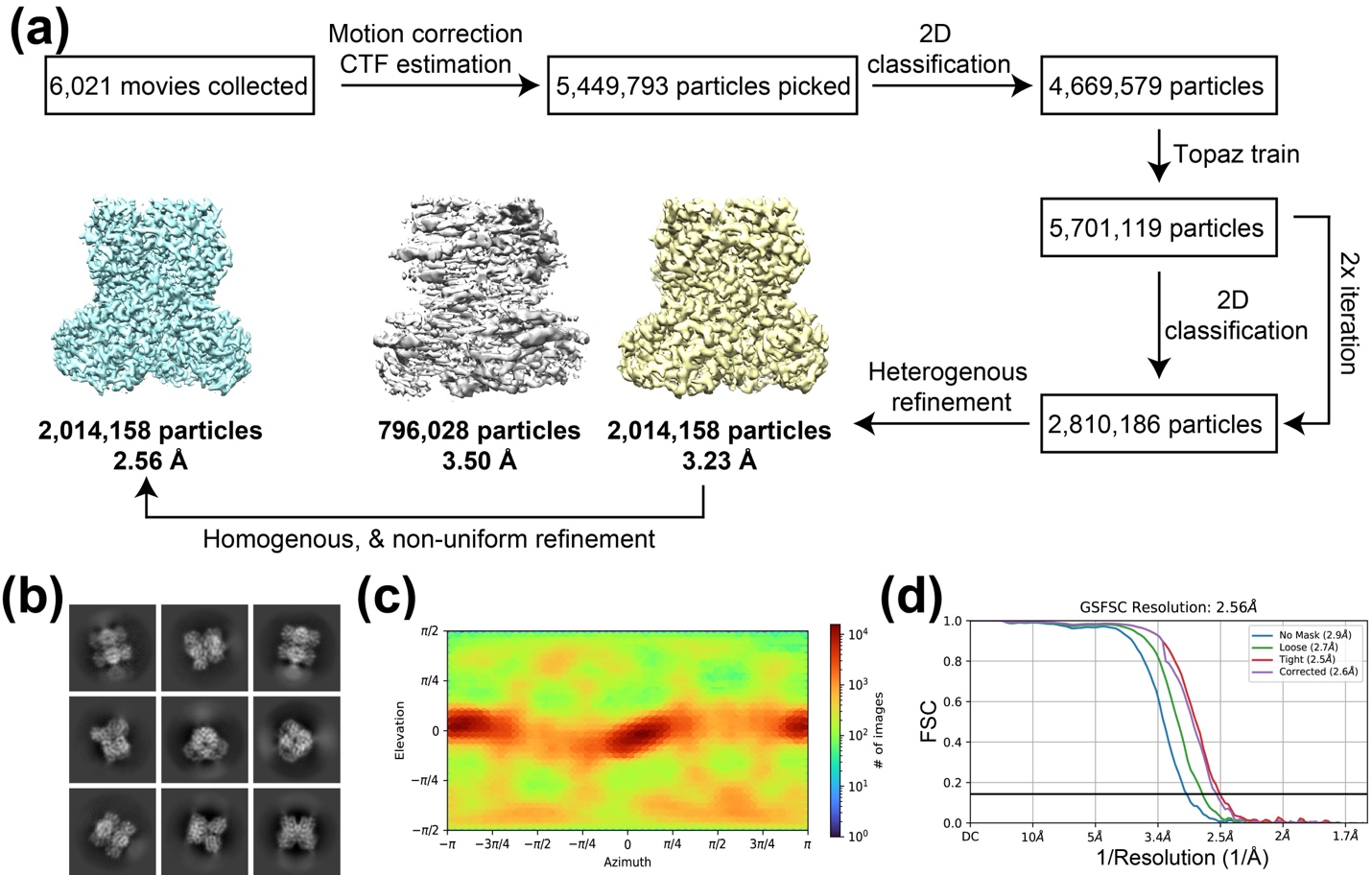


Figure S3. Workflow for cryo-EM data processing for G3P bound GapN. A) Workflow of cryo-EM data processing for the substrate bound GapN. B) Representative 2D class averages of picked particles collected from substrate bound GapN. C)Angular distribution heat map of particle projections for the G3P bound GapN. E) Gold standard Fourier shell correlation (FSC) curve for G3P bound GapN reconstructions. Solid line represents the overall nominal resolution of each reconstruction at 0.143 FSC calculated by CryoSPARC.

Table S1. Data collection and model refinement of Apo GAPN, the GAPN/NADP+ complex, and the GAPN/G3P complex.

|  | Apo GAPN | GAPN/ NADP^+^ | GAPN/G3P |
| --- | --- | --- | --- |
| Data Collection and Processing | | | |
| Microscope | Titan Krios | Titan Krios | Titan Krios |
| Voltage (kV) | 300 | 300 | 300 |
| Magnification | 29,000 | 29,000 | 29,000 |
| Electron Dose (e-/$Å$^2^) | 49 | 50 | 50 |
| Camera | Gatan K3 | Gatan K3 | Gatan K3 |
|  |  |  |  |
| Defocus range (um) | -0.8 to -2.3 | -0.8 to -2.2 | -0.8 to -2.2 |
| Pixel size ($Å$) super resolution | 0.506 | 0.394 | 0.394 |
| Movies collected | 6,756 | 5,368 | 6,021 |
| Symmetry imposed | C2 | C2 | C1 |
| Final particle images (no.) | 2,301,844 | 1,068,956 | 2,014,158 |
| Map resolution ($Å$) | 2.71 | 2.82 | 2.56 |
| Sharpening B-factor ($Å$^2^) | 149.83 | 141.21 | 119.74 |
| Software used to process data | cryoSPARC | cryoSPARC | cryoSPARC |
| Refinement statistics |  |  |  |
| Number of protein atoms (non-H) | 14375 | 14567 | 14415 |
| R.m.s deviations |  |  |  |
| Bond lengths ($Å$) | 0.01 | 0.007 | 0.01 |
| Bond angles (°) | 1.121 | 0.814 | 0.945 |
| Validation |  |  |  |
| MolProbity score | 2.03 | 2.01 | 2.39 |
| Clash score | 11.2 | 9.97 | 10.53 |
| Poor rotamers (%) | 1.41 | 3.21 | 1.34 |
| Ramanchandram plot |  |  |  |
| Favored (%) | 94.91 | 97.45 | 95.65 |
| Allowed (%) | 5.04 | 2.55 | 4.35 |
| Disallowed (%) | 0.05 | 0 | 0 |
| C-beta deviations | 0.12 | 0 | 0 |
| Model vs Data CC | 0.76 | 0.71 | 0.66 |
| FSC model (0.143) | 2.6 | 2.8 | 2.4 |
| EMDB access code | EMD-46862 | EMD-46868 | EMD-46869 |
| PDB access code | 9DLB | 9DLA | 9DLC |
